# Correction: Discovery of fully synthetic FKBP12-mTOR molecular glues

**DOI:** 10.1039/d6sc90062c

**Published:** 2026-03-23

**Authors:** Robin C. E. Deutscher, Christian Meyners, Maximilian L. Repity, Wisely Oki Sugiarto, Jürgen M. Kolos, Edvaldo V. S. Maciel, Tim Heymann, Thomas M. Geiger, Stefan Knapp, Frederik Lermyte, Felix Hausch

**Affiliations:** a Institute for Organic Chemistry and Biochemistry, Technical University Darmstadt Peter-Grünberg-Straße 4 64287 Darmstadt Germany felix.hausch@tu-darmstadt.de; b Institut für Pharmazeutische Chemie, Goethe-University Frankfurt Biozentrum, Max-von-Laue-Str. 9 60438 Frankfurt am Main Germany; c Structural Genomics Consortium, Goethe-University Frankfurt, Buchmann Institute for Life Sciences Max-von-Laue-Str. 15 60438 Frankfurt am Main Germany; d German Cancer Consortium (DKTK)/German Cancer Research Center (DKFZ) DKTK Site Frankfurt-Mainz 69120 Heidelberg Germany; e Centre for Synthetic Biology, Technical University of Darmstadt 64287 Darmstadt Germany

## Abstract

Correction for ‘Discovery of fully synthetic FKBP12-mTOR molecular glues’ by Robin C. E. Deutscher *et al.*, *Chem. Sci.*, 2025, **16**, 4256–4263, https://doi.org/10.1039/D4SC06917J.

The authors regret that there is an error in Table 1 of the article, within the heading of the fifth column. The units for EC^ternary nanoBRET^_50_ should be nM. Furthermore, a citation to ref. 58 should be present. The correct version of Table 1 is displayed below.

**Table 1:** Biochemical and cellular characterization of FKBP12-FRB molecular glues. Affinities for compounds binding to purified human FKBP12 were determined by a competitive FP assay (*K*^FP^_i_).^56^ Biochemical potencies for ternary complex induction were determined using a FP assay by titrating purified FRB with compound-bound fluorescently labelled FKBP12 (EC^ternary FP^_50_). Intracellular potencies for FKBP12 occupancy were determined by a competitive NanoBRET assay (IC^nanoBRET^_50_)^57^ and potencies for intracellular formation of FKBP12-compound-FRB ternary complexes were determined by HEK293T cells transiently expressing a FKBP12-nLuc and FRB-HaloTag BRET pair (EC^ternary nanoBRET^_50_). n.b. = non-binding, n.m. = not measured

No.	Human FKBP12, *K*^FP^_i_/nM^56^	EC^ternary FP^_50_/µM	FKBP12 IC^NanoBRET^_50_/nM^57^	EC^ternary NanoBRET^_50_/nM^58^	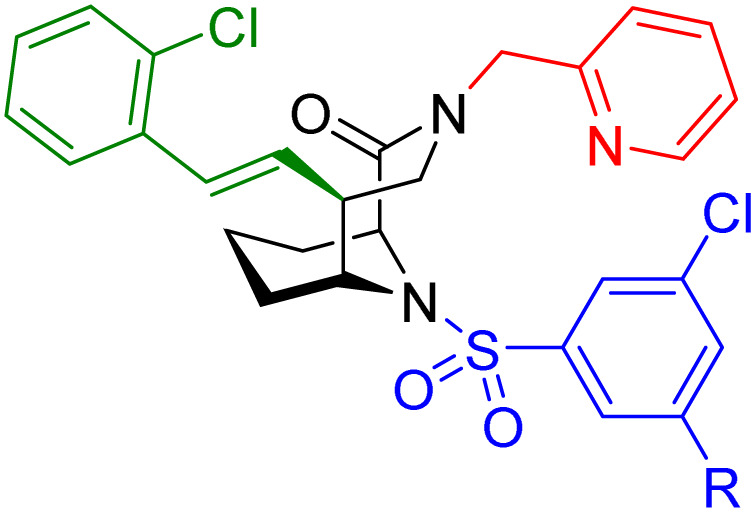
Rapamycin 1	0.6	0.039 ± 0.006	30.3 ± 1.5	1.8 ± 0.16	—
7	6.3	93 ± 21	81.2 ± 16.3	n.b.	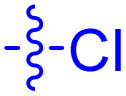
9a	5.8	56 ± 10	40.6 ± 5.3	n.m.	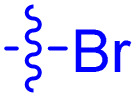
9b	3.6	54 ± 6	47.8 ± 10.7	n.m.	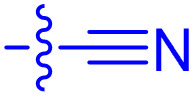
10a	11	50 ± 5	405 ± 219	n.b.	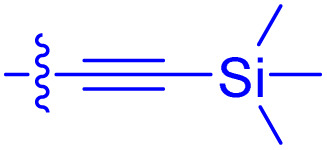
10b	13	63 ± 6	101 ± 19	n.m.	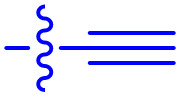
10c	5.1	7.8 ± 2.6	146 ± 28.5	n.b.	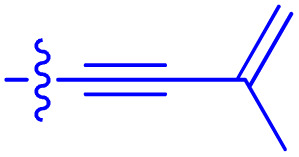
10d	4.5	4.1 ± 0.4	25.5 ± 3.0	50.5 ± 9.8	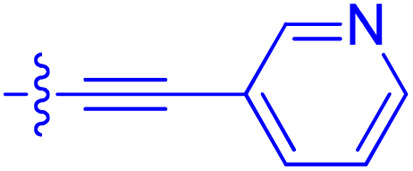
10e	6.9	2.0 ± 0.2	57.3 ± 16.8	38.8 ± 1.7	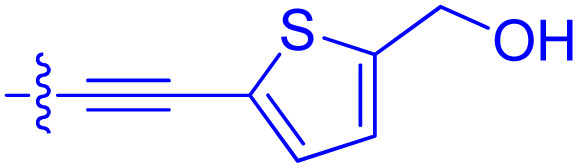
10f	1.8	1.9 ± 0.2	259 ± 37	28.2 ± 1.3	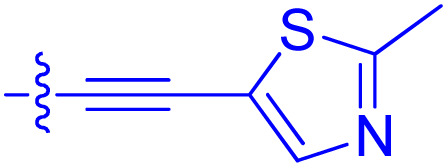
10g	4.7 ± 1.8	1.8 ± 0.1	49.2 ± 4.0	26.0 ± 1.6	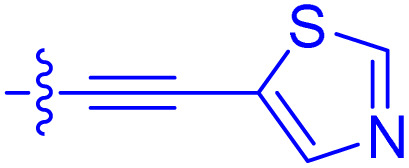
10h	0.8	1.5 ± 0.2	66.9 ± 22.5	31.7 ± 2.5	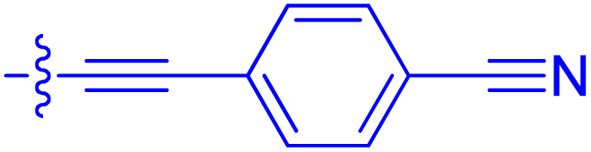
10i	0.4	1.3 ± 0.2	264 ± 36.8	172 ± 36	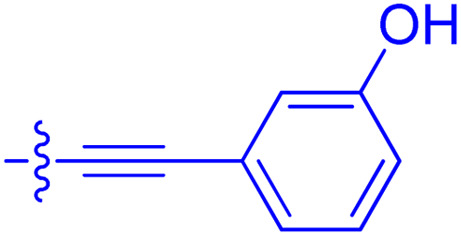
10j	7.2 ± 1.7	0.63 ± 0.06	799 ± 183	57.5 ± 3.6	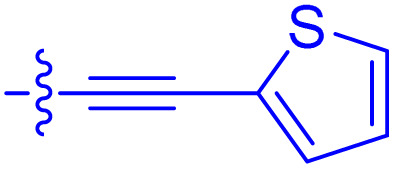
10k	4.1 ± 0.6	0.56 ± 0.03	314 ± 21	26.3 ± 1.3	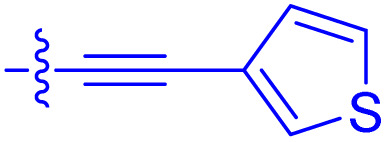
10l	4.5	0.53 ± 0.07	527 ± 77	32.9 ± 2.5	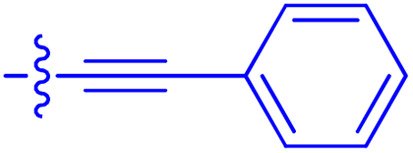
10m	6.0	0.23 ± 0.03	952 ± 147	31.7 ± 2.1	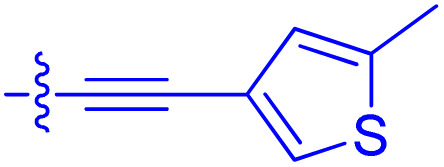
10n	4.8 ± 0.8	0.18 ± 0.02	1330 ± 195	42.1 ± 2.8	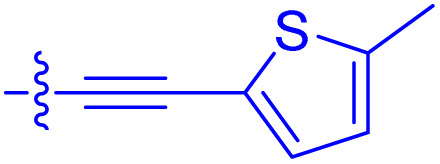
10o	2.6	0.17 ± 0.02	7460 ± 2100	109 ± 6.3	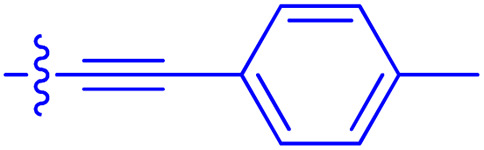

The Royal Society of Chemistry apologises for these errors and any consequent inconvenience to authors and readers.

